# Polymorphisms of *HIF1A* gene are associated with prognosis of early stage non-small-cell lung cancer patients after surgery

**DOI:** 10.1007/s12032-014-0877-8

**Published:** 2014-02-25

**Authors:** Boya Liu, Qingchun Liu, Yang Song, Xiaofei Li, Yunjie Wang, Shaogui Wan, Zhipei Zhang, Haichuan Su

**Affiliations:** 1 Department of Thoracic Surgery, Tangdu Hospital, The Fourth Military Medical University, 569 Xinsi Road, Xi’an, 710038 China; 2 Department of Thoracic Surgery, Hanzhong Central Hospital, Hanzhong, 723000 China; 3 Department of Oncology, Tangdu Hospital, The Fourth Military Medical University, 569 Xinsi Road, Xi’an, 710038 China; 4 Pharmaceutical College of Henan University, Kaifeng, 475001 Henan China

**Keywords:** *HIF1A*, Single nucleotide polymorphism, Association study, Non-small-cell lung cancer

## Abstract

Hypoxia-inducible factor 1α (HIF1α) activates the transcription of genes that are involved in angiogenesis and cell survival. Over-expression of HIF1α caused by intratumoral hypoxia and its genetic alterations are associated with increased mortality in several cancer types including non-small-cell lung cancer (NSCLC). The aim of this study was to investigate the predictive role of single nucleotide polymorphisms (SNPs) in *HIF1A* gene in NSCLC outcomes. We genotyped two functional SNPs (rs2057482 and rs2301113) in *HIF1A* gene and assessed their associations with clinicopathological parameters and prognosis of 494 NSCLC patients by Cox proportional hazard model. There was no significant association between the SNPs and clinical outcomes of NSCLC for overall analysis. However, in stratified analysis for NSCLC patients at early stage (I/II), we observed a protective effect conferred by variant genotype of rs2057482 on overall survival (OS) (HR 0.42, 95 % CI 0.22–0.80) and recurrence-free survival (RFS) (HR 0.60, 95 % CI 0.36–0.97) in a dominant model. Additionally, multivariate Cox analysis based on dominant model indicated that significant increased death and recurrence risks were observed in patients with early T*-*stage (T1 and T2) tumors, who carrying variant-containing genotype of rs2301113, as well as in patients without lymph node involvement (N0 stage) for rs2057482. Genetic variations on *HIF1A* gene are significantly associated with NSCLC outcomes in patients with early stage disease.

**Electronic supplementary material:**

The online version of this article (doi:10.1007/s12032-014-0877-8) contains supplementary material, which is available to authorized users.

## Introduction

Lung cancer is the leading cause of cancer-related death worldwide. Non-small-cell lung cancer (NSCLC) represents 75–85 % of all lung cancer cases. The median survival time of patients with untreated metastatic NSCLC is only 4–5 months, with a 1-year survival rate of only 10 % [1, 2]. Up to date, the prediction of NSCLC prognosis largely depends on the conventional prognostic factors such as disease stage and performance status. NSCLC patients with the same pathological features, however, have dramatically distinct response to chemotherapies and have different survival outcomes, suggesting the importance of novel biomarkers for the prognosis of NSCLC [3].

Hypoxia-inducible factor-1 (HIF1) is a key regulator of cellular response to hypoxia and plays essential roles in regulating angiogenesis, cell adhesion, energy metabolism, and apoptosis [4–8]. HIF1 is a heterodimeric transcriptional complex consisting of *α* and *β* subunits, in which HIF1α is the oxygen-regulated factor that determines the activity of HIF1 [9]. Since hypoxia-induced angiogenesis is required for tumor growth, HIF1α is heavily involved in the development and progression of several types of human malignancies [10–13]. For example, overexpression of HIF1α increased the invasive capacity of human lung adenocarcinoma cells [14], while antisense oligonucleotide of HIF1α inhibited the proliferation of lung cancer cells both in vitro and in vivo [15]. Besides, the expression of HIF1α is associated with the prognosis of cancer patients. For example, lung squamous cell carcinoma patients with high HIF1α expression in tumor cells and low expression in stromal cells had better survival [16]. In lung adenocarcinoma, expression of HIF1α was significantly higher in cases with vascular invasion, lymph node involvement, and vascular endothelial growth factor-A expression [17]. Similarly, the expression level of HIF1α was significantly higher in tumor tissue than in the corresponding non-tumor lung tissue and was associated with a shorter survival time in NSCLC [16, 18–20]. In contrast, patients with HIF-positive carcinomas had significantly longer median survival time than patients with HIF-negative carcinomas [21]. All these data strongly support the hypothesis that the presence of HIF1 may serve as a prognostic biomarker for the survival of NSCLC patients.

The *HIF1A* gene is highly polymorphic, and its expression and activity are dramatically affected by various polymorphisms. In addition to its expression, polymorphisms of *HIF1A* have also been implicated in the carcinogenesis of several malignancies. For example, two single nucleotide polymorphisms (SNPs) rs11549465 and rs11549467 on *HIF1A* locus, which resulted in proline-to-serine and alanine-to-threonine amino acid substitutions, respectively, were found to be associated with unfavorable prognosis in oral squamous cell carcinoma [22]. When it came to cancer risks, there was a significant association between rs11549465 and the risk of developing colorectal cancer [23], and the combined variant genotypes of rs2057482 and rs11549467 were associated with increased prostate cancer risk [24]. In addition, rs11549467 has been involved in the progression of pancreatic ductal adenocarcinoma [25]. Given the important role of HIF1α in the progression of NSCLC, it is plausible that polymorphisms of *HIF1A* may affect the biological behavior and prognosis of NSCLC. Up to date, however, there is no report on the role of *HIF1A* polymorphism in NSCLC prognosis. In this study, we for the first time investigated the association between *HIF1A* polymorphisms and prognosis of NSCLC in our ongoing hospital-based cohort study in a Chinese Han population. We assessed the effects of 2 functional SNPs in *HIF1A* on recurrence and survival in 494 NSCLC patients undergoing surgical treatment.

## Materials and methods

### Study population

A total of 564 patients with incident NSCLC were initially recruited into an ongoing molecular epidemiological study at the Department of Thoracic Surgery of Tangdu Hospital, the Fourth Military Medical University, Xi’an, China from July 2009 to December 2011. There was no restriction for case recruitment. All patients were newly diagnosed and histologically confirmed with NSCLC. In the present study, we excluded 70 patients, including 17 patients who did not undergo surgery or only received palliative operation, 38 patients who had incomplete clinical information, 6 patients who were not Han Chinese, 6 patients died within 2 months after surgery, 3 patients had relapsed within 1 month, and finally, 494 patients were included in this study. This study was approved by the Ethic Committee of the Fourth Military Medical University, and all experimental procedures were performed according to Helsinki Declaration. Signed informed consent was obtained from each participant.

### Demographic and clinical data

Demographic data were collected through in-person interviews using a standardized epidemiological questionnaire, including age, gender, ethnicity, residential region, smoking status, alcohol use, education status, and family history of cancer. Detailed clinical information was collected through medical chart review or consultation with treating physicians, including time of diagnosis, time of surgery and/or chemotherapies, time of recurrence and/or death, tumor stage, differentiation, location site, lymph node invasiveness, and treatment protocol. The tumor histological grade was assessed according to WHO criteria, and tumors were staged using the 7th edition TNM staging system based on post-operative pathological examination of the specimens after surgery. A standard follow-up was performed by a trained clinical specialist through on-site interview, direct calling, or medical chart review. The latest follow-up data in this analysis were obtained in August 2012, and 37 (6.56 %) patients were lost during follow-up. For each patient enrolled, 5 ml of venous blood was available for genomic DNA extraction using the E.Z.N.A. Blood DNA Midi Kit (Omega Bio-Tek, Norcross, GA) in the laboratory.

### SNP selection and genotyping

Functional SNPs in *HIF1A* gene were selected using a set of Web-based SNP selection tools (freely available at http://snpinfo.niehs.nih.gov/snpinfo/snpfunc.htm), by which one can select SNPs based on linkage disequilibrium and predict functional characteristics of both coding and non-coding SNPs. The 5′- and 3′-flanking regions were arbitrarily set at 1,000 bp for all genes. Only validated SNPs were selected, and SNPs with minor allele frequency <5 % in the Asian population were excluded. In the case of multiple potentially functional SNPs within the same haplotype block (defined by the linkage coefficient *r* ^2 ^> 0.8), none of SNPs was included. Functional SNPs include non-synonymous SNPs in exons, SNPs in miRNA binding sites of 3′-untranslated region (UTR), SNPs in the transcription factor binding site of the 5′ flanking region, and SNPs in splice sites. Finally, we found only one functional SNP locus (rs2057482), which located in the 3′-UTR. Another SNP locus, rs2301113, was selected as previous studies reported [26, 27]. Genotyping was performed using Sequenom iPLEX genotyping system (Sequenom Inc, CA). Laboratory personnel conducting genotyping was blinded to patient information. The average call rate for the SNP array was 99.5 %. Strict quality control measures were implemented during genotyping with >99.0 % concordance with the main genotyping results.

### Statistical analysis

SPSS19.0 statistics software was used for all statistical analyses. Two major endpoints were analyzed in this study, which were overall survival (OS) and recurrence-free survival (RFS). OS time was defined as the date from primary NSCLC diagnosed to the date of death from any causes. RFS time was defined as the date from cancer diagnosis to the date of the first time disease recurrence. Patients who were still alive at the last contact date were censored for the OS analysis, and those who did not develop recurrence at the last contact date were censored for the RFS analysis. The three genetic models (additive, dominant, and recessive) were applied to assess the association of single SNPs with clinical outcomes of NSCLC patients. Considering that there was a very small number (≤5) of patients with the rare homozygous variant genotype for 2 SNPs of *HIF1A* gene, we did not analyze the recessive model for these 2 SNPs. Hazards ratios (HRs) and 95 % confidence intervals (CIs) were estimated by a multivariate Cox proportion hazards model, adjusting for age, gender, smoking status, histology, TNM stage, differentiation, and adjuvant therapy. A powerful bootstrap resampling was performed 100 times to reduce the potential spurious findings. Kaplan–Meier curve and log-rank test were used to assess the differences of patient groups with different genotypes for OS and RFS. All *P* values in this study were two-sided. *P* < 0.05 was considered as statistical significant.

## Results

### Distribution of patients characteristics and prognosis analysis

Characteristics of 494 NSCLC patients were summarized in Table 1. Among these 494 patients, the median age at the time of diagnosis was 59 years (ranging from 27 to 86 years), and 385 (77.9 %) were males. There were 270 patients diagnosed with squamous cell carcinoma, 149 with adenocarcinoma, and 75 with other types of NSCLC, including 55 with adenosquamous carcinoma, 15 with carcinosarcoma, 3 with large-cell lung cancer, and 2 with mucoepidermoid carcinoma. The pathological stages were as follows: 287 at stage I/II (58.1 %) and 207 at stage III (41.9 %). The majority of patients (69.8 %) had well or moderately differentiated tumors, and 365 (73.9 %) patients had received adjuvant chemotherapy or radiotherapy after surgery. In the follow-up period of 20.3 months, 149 (30.2 %) patients died and 209 (42.3 %) patients developed recurrence.

**Table 1:** Distribution of patients’ characteristics and prognosis analysis

Variable	No. of total patients (%)	Overall survival			Recurrence-free survival		
		No. of deaths (%)	HR (95 % CI)^a^	*P* value	No. of recurrences (%)	HR (95 % CI)^a^	*P* value
Age							
≤59	244 (49.4)	71 (47.7)	Ref.		110 (52.6)	Ref.	
>59	250 (50.6)	78 (52.3)	0.89 (0.63–1.25)	0.503	99 (47.4)	0.84 (0.63–1.11)	0.217
Gender							
Female	109 (22.1)	32 (21.5)	Ref.		50 (23.9)	Ref.	
Male	385 (77.9)	117 (78.5)	1.07 (0.61–1.87)	0.826	159 (76.1)	1.16 (0.72–1.87)	0.532
Smoking status							
Never smoker	154 (31.2)	45 (30.2)	Ref.		70 (33.5)	Ref.	
Ever smoker	340 (68.8)	104 (69.8)	0.94 (0.57–1.56)	0.812	139 (66.5)	0.77 (0.50–1.17)	0.224
Histology							
Squamous cell carcinoma	270 (54.7)	79 (53.0)	Ref.		109 (52.2)	Ref.	
Adenocarcinoma	149 (30.2)	33 (22.1)	0.59 (0.38–0.92)	0.020	57 (26.8)	0.74 (0.52–1.06)	0.108
Others^b^	75 (15.1)	37 (24.8)	1.54 (0.91–2.59)	0.106	44 (21.1)	1.22 (0.78–1.93)	0.375
TNM Stage							
I/II	287 (58.1)	74 (48.7)	Ref.		98 (46.9)	Ref.	
III	207 (41.9)	78 (51.3)	1.85 (1.33–2.57)	<0.001	111 (53.1)	1.94 (1.47–2.56)	<0.001
T-stage							
T1/2	306 (61.9)	80 (53.7)	Ref.		117 (56.0)	Ref.	
T3/4	188 (38.1)	69 (46.3)	1.41 (0.98–2.05)	0.067	92 (44.0)	1.24 (0.90–1.70)	0.184
N-stage							
N0	231 (46.8)	60 (40.3)	Ref.		77 (36.8)	Ref.	
N1/2/3	263 (53.2)	89 (59.7)	1.45 (0.87–2.44)	0.161	132 (63.2)	1.60 (1.05–2.46)	0.032
Differentiation							
Well/moderate	345 (69.8)	85 (57.0)	Ref.		125 (59.8)	Ref.	
Poorly/undifferentiated	149 (30.2)	64 (43.0)	1.35 (0.89–2.07)	0.163	84 (40.2)	1.56 (1.10–2.22)	0.013
Adjuvant therapy							
No	129 (26.1)	45 (30.2)	Ref.		52 (24.9)	Ref.	
Yes	365 (73.9)	104 (69.8)	0.44 (0.31–0.63)	<0.001	157 (75.1)	0.72 (0.53–0.99)	0.040

*CI* confidence interval, *HR* hazard ratio, *Ref* reference

^a^HRs were adjusted for age, gender, smoking status, histology, TNM stage, differentiation and adjuvant chemotherapy or radiotherapy, where appropriate

^b^Other carcinomas include adenosquamous carcinoma, large cell carcinoma, carcinosarcoma, and mucoepidermoid carcinoma

Multivariate Cox regression analyses were performed to assess the prognostic effects of clinical characteristics such as age, gender, smoking status, histology, TNM stage, differentiation grade, and adjuvant therapy (Table 1). Our analyses showed that patients with advanced TNM stage (stage III) had significant higher death risk (HR 1.85, 95 % CI 1.33–2.57) and recurrence risk (HR 1.94, 95 % CI 1.47–2.56), comparing to those with early TNM stages (I + II) for OS and RFS, respectively. As expected, patients who received adjuvant chemotherapy or radiotherapy exhibited significantly reduced death risk (HR 0.44, 95 % CI 0.31–0.63) and recurrence risk (HR 0.72, 95 % CI 0.57–0.99), comparing to those without adjuvant therapy for OS and RFS, respectively. Additionally, significantly increased recurrence risks were also observed in patients with lymph nodes involvement (*P* = 0.032) or poorly and undifferentiated tumors (*P* = 0.013).

### Association of *HIF1A* SNPs with clinical outcomes in NSCLC patients

**Fig. 1:**
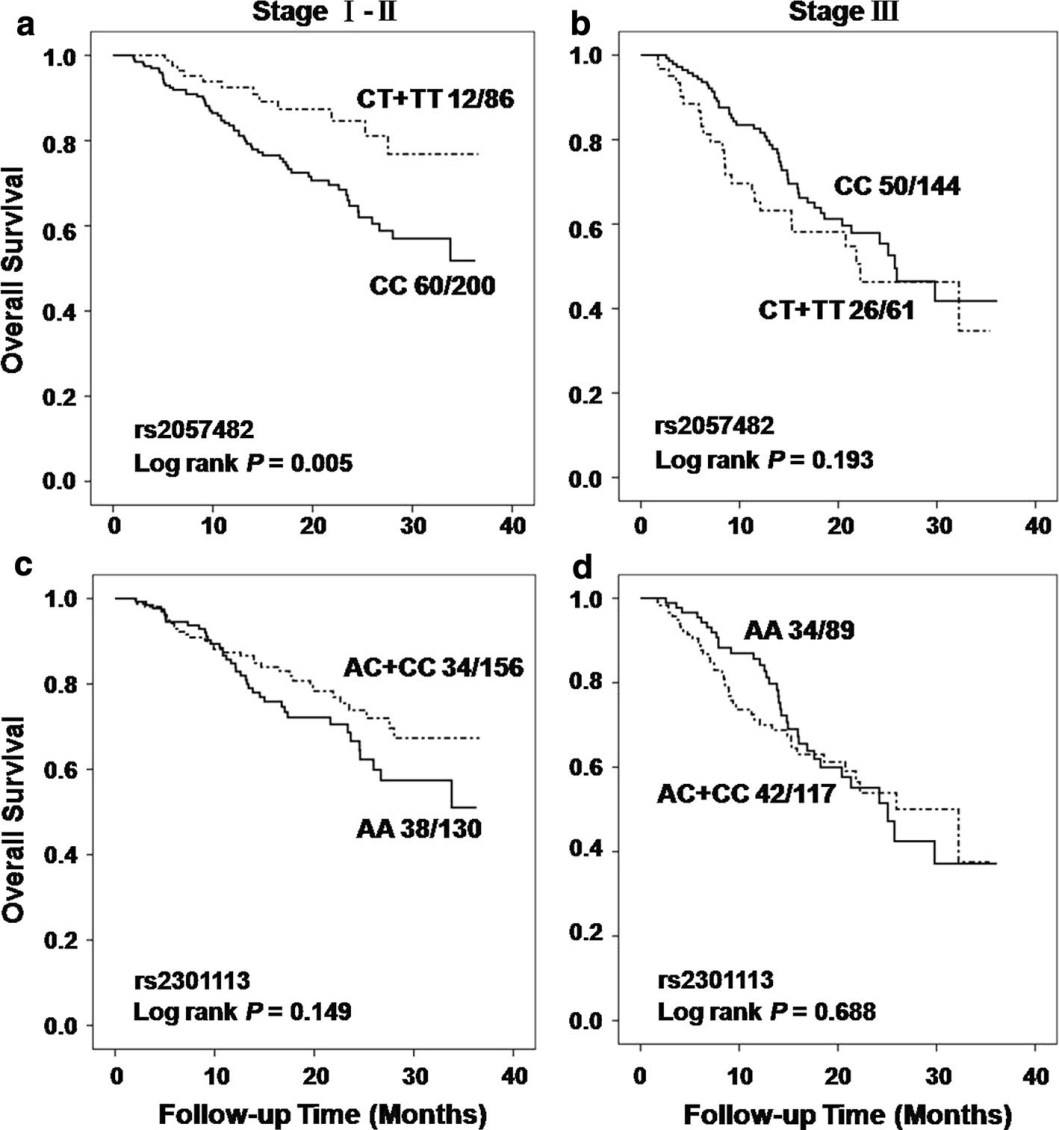
Kaplan–Meier curves of overall survival by dominant model in patient subgroups with different disease stages. **a** The analysis of rs2057482 in patients with early stage disease; **b** rs2057482 in patients with advanced stage disease; **c** rs2301113 in patients with early stage disease; **d** rs2301113 in patients with advanced stage disease

We assessed the effect of 2 SNPs (rs2057482 and rs2301113) in *HIF1A* gene on death and recurrence in NSCLC patients using a multivariate Cox regression model. However, there was no significant association observed between the two polymorphisms and OS or RFS in the analysis (Online resource Table 1). We then performed a stratified analysis to evaluate OS and RFS in different strata of patients’ characteristics in NSCLC patients. Surprisingly, as shown in online resource Table 2 and Table 2, significant protective effects of variant-containing genotypes of rs2057482 were observed in patients with early stage (TNM stage I + II) for both OS (HR 0.42, 95 % CI 0.22–0.80) and RFS (HR 0.60, 95 % CI 0.36–0.97) analyses, when comparing to those carrying wild-type genotype. To internally validate the results, we next performed random bootstrap sampling of the significant SNP (rs2057482) for 100 iterations and listed the number of times that the *P* value was <0.05. In early stage (I + II) patients, highly consistent results were observed, with bootstrap *P* < 0.05 for 100 of the sampling in OS and 62 of the sampling in RFS. We also observed significant associations between variant-containing genotypes of both SNPs and reduced recurrence risk in patients with well and moderate differentiated tumors, when comparing to those carrying wild-type genotype. The Kaplan–Meier curve analyses consistently showed that in early stage (I + II), NSCLC patients who carrying variant-containing genotypes of rs2057482 had significant longer overall survival time (log rank *P* = 0.005) (Fig. 1) and time to recurrence (log rank *P* = 0.047) (Online resource Fig 1), when comparing to those carrying wild-type genotype. Since tumor stage is the most effective prognostic factor in predicting cancer patients’ survival, we further focused the analyses of SNPs and NSCLC patients’ outcomes by different stage diseases.

**Table 2:** Associations between SNPs and NSCLC outcomes in subgroups of patients with different disease stages

SNP	Patient subgroup	Genotypes	Overall survival				Recurrence-free survival			
			Death/total	HR (95 % CI)^a^	*P* value	No. times in bootstrap^b^ sample *P* < 0.05	Recurrence/total	HR (95 % CI)^a^	*P* value	No. times in bootstrap^b^ sample *P* < 0.05
rs2057482	Stage I+II	CC	60/200	Ref.			75/200	Ref.		
		CT+TT	12/86	0.42 (0.22–0.80)	0.008	100	22/86	0.60 (0.36–0.97)	0.039	62
	Stage III	CC	50/144	Ref.			79/144	Ref.		
		CT+TT	26/61	1.54 (0.94–2.53)	0.087		31/61	0.93 (0.60–1.44)	0.735	
rs2301113	Stage I+II	AA	38/130	Ref.			49/130	Ref.		
		AC+CC	34/156	0.71 (0.44–1.15)	0.165		48/156	0.77 (0.51–1.16)	0.206	
	Stage III	AA	34/89	Ref.			52/89	Ref.		
		AC+CC	42/117	1.04 (0.66–1.65)	0.866		58/117	0.89 (0.61–1.32)	0.569	

^a^HRs were adjusted for age, gender, smoking status, histology, differentiation and adjuvant chemotherapy or radiotherapy

^b^Non-significant SNPs were not tested using the bootstrapping method

### Association of *HIF1A* SNPs with clinical outcomes in different T-stage or N-stage disease

**Fig. 2:**
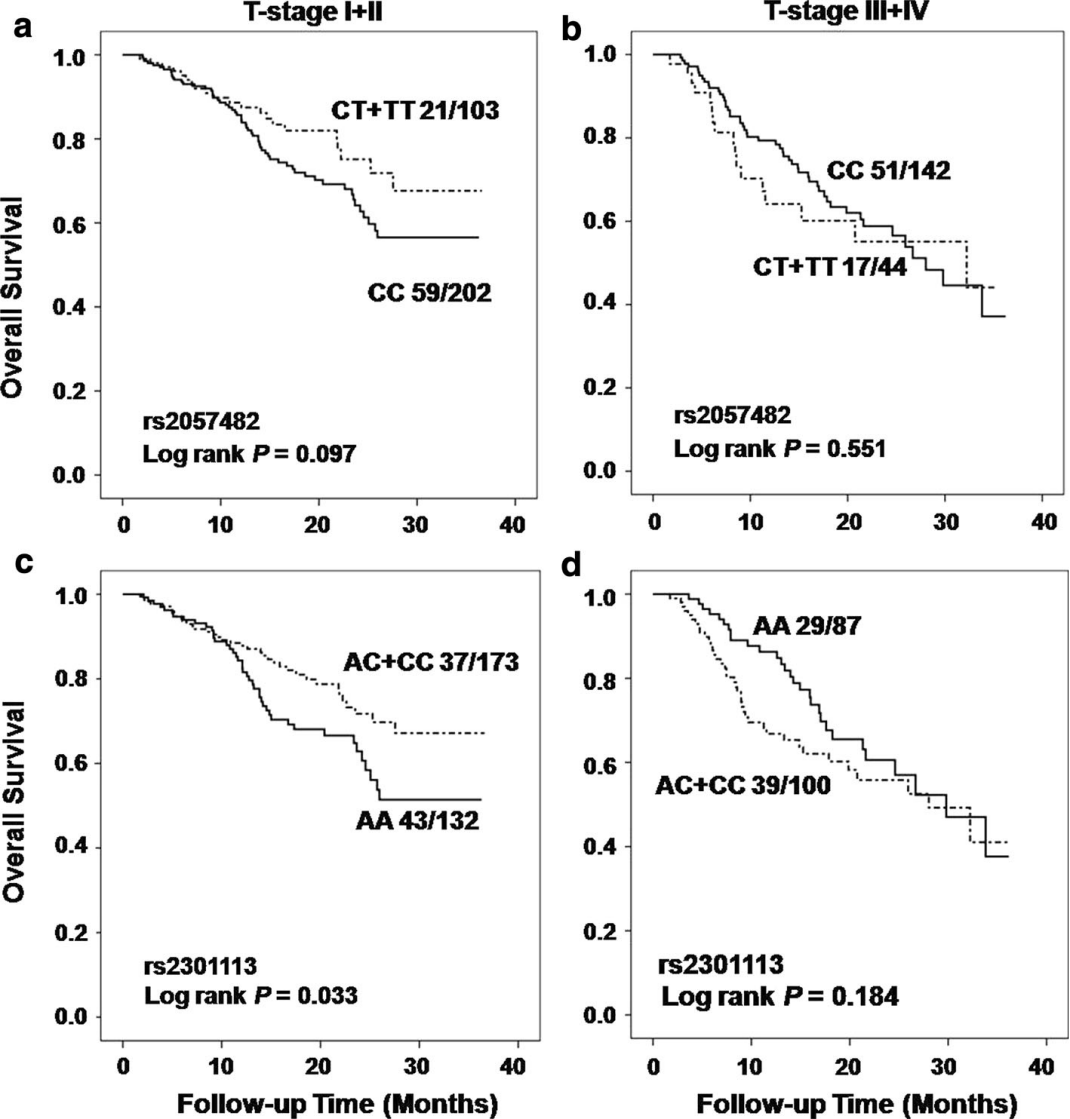
Kaplan–Meier curves of overall survival by dominant model in patient subgroups with different T-stages. **a** The analysis of rs2057482 in patients with early T-stage disease; **b** rs2057482 in patients with advanced T-stage disease; **c** rs2301113 in patients with early T-stage disease; **d** rs2301113 in patients with advanced T-stage disease

**Fig. 3:**
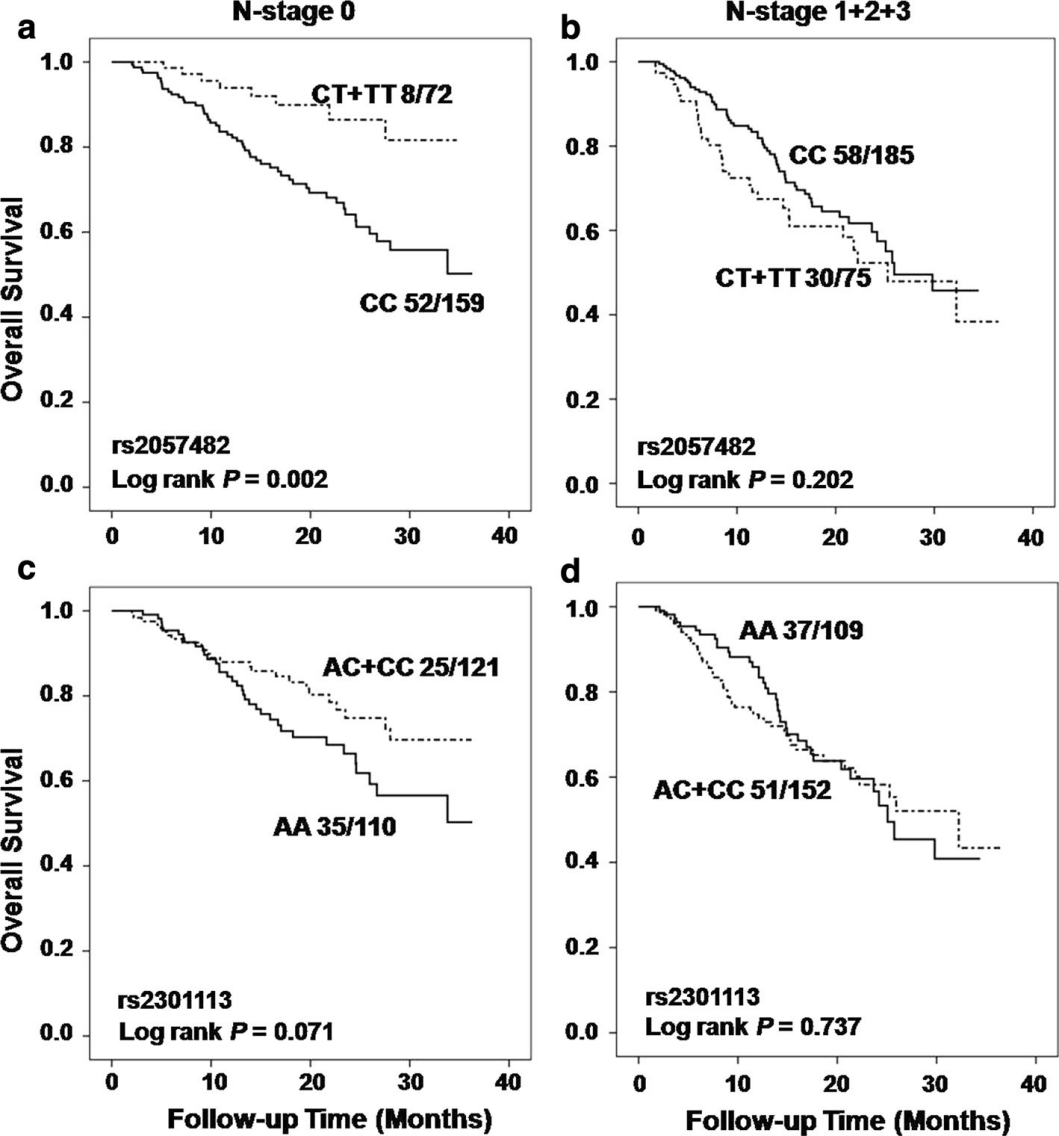
Kaplan–Meier curves of overall survival by dominant model in patient subgroups with different N-stages. **a** The analysis of rs2057482 in patients without lymph node involvement; **b** rs2057482 in patients with positive lymph nodes involvement; **c** rs2301113 in patients without lymph node involvement; **d** rs2301113 in patients with positive lymph nodes involvement

We further analyzed the associations between the SNPs and more detailed tumor stages of T-stage (tissue invasion) or N-stage (lymph nodes involvement), and stratified the analyses by stage status. As the data shown in Table 3, the OS analysis results of significant associations between reduced death risks and variant-containing genotypes of SNP rs2057482 (HR 0.60, 95 % CI 0.36–1.00, *P* = 0.050) and rs2301113 (HR 0.60, 95 % CI 0.38–0.93, *P* = 0.023) were consistently observed in patients with early T-stage (I + II), instead of in patients with advanced T-stages, when comparing to those carrying wild-type genotype. Similarly, for RFS analysis, we also observed the significant associations between variant-containing genotypes of rs2057482 or rs2301113 and reduced recurrence risk with HR of 0.59 (95 % CI 0.39–0.90, *P* = 0.015) and 0.61 (95 % CI 0.42–0.89, *P* = 0.010), respectively, in patients with early T-stage diseases (Table 3). Moreover, the variant-containing genotypes of rs2053482 exhibited significant associations with reduced death risk (HR 0.34, 95 % CI 0.16–0.73, *P* = 0.005) and recurrence risk (HR 0.49, 95 % CI 0.27–0.86, *P* = 0.014) in patients without lymph node involvement (N0 stage patients), comparing to those carrying wild-type genotype (Table 3). Attenuated similar results were also observed in the same analyses for SNP rs2301113 with borderline significance in OS (*P* = 0.063) and RFS (*P* = 0.084) analyses, in patients without lymph node involvement (Table 3). The Kaplan–Meier curve analyses indicated that variant-containing genotypes of rs2301113 significantly distinguished patients with better survival in OS and RFS analyses from those carrying wild-type genotype with log rank *P* value of 0.033 and 0.015, respectively, in patient group with early T-stage, instead of in patient group with advanced T-stage disease (Fig. 2 for OS and Online resource Fig. 2 for RFS). While for SNP rs2057482, the variant-containing genotypes significantly distinguished patients with better survival in patients without lymph node involvement in OS and RFS analyses with log rank *P* value of 0.002 and 0.010, respectively (Fig. 3 for OS and Online resource Fig 3 for RFS).

**Table 3:** Associations between SNPs and NSCLC outcomes in subgroups of patients with different T-stage or N-stage disease

SNP	Variables	Genotypes	Overall survival			Recurrence-free survival		
			Death/total	HR (95 % CI)^a^	*P* value	Recurrence/total	HR (95 % CI)^a^	*P* value
rs2057482	T1+T2	CC	59/202	Ref.		85/202	Ref.	
		CT+TT	21/103	0.60 (0.36–1.00)	0.050	31/103	0.59 (0.39–0.90)	0.015
	T3+T4	CC	51/142	Ref.		69/142	Ref.	
		CT+TT	17/44	1.39 (0.78–2.46)	0.263	22/44	1.15 (0.70–1.91)	0.576
	N0	CC	52/159	Ref.		62/159	Ref.	
		CT+TT	8/72	0.34 (0.16–0.73)	0.005	15/72	0.49 (0.27–0.86)	0.014
	N1+N2+N3	CC	58/185	Ref.		92/185	Ref.	
		CT+TT	30/75	1.34 (0.85–2.11)	0.209	38/75	1.02 (0.69–1.51)	0.921
rs2301113	T1+T2	AA	43/132	Ref.		61/132	Ref.	
		AC+CC	37/173	0.60 (0.38–0.93)	0.023	55/173	0.61 (0.42–0.89)	0.010
	T3+T4	AA	29/87	Ref.		40/87	Ref.	
		AC+CC	39/100	1.37 (0.84–2.26)	0.210	51/100	1.34 (0.88–2.04)	0.176
	N0	AA	35/110	Ref.		43/110	Ref.	
		AC+CC	25/121	0.61 (0.36–1.03)	0.063	34/121	0.67 (0.42–1.06)	0.084
	N1+N2+N3	AA	37/109	Ref.		58/109	Ref.	
		AC+CC	51/152	1.02 (0.67–1.57)	0.922	72/152	0.93 (0.66–1.33)	0.697

^a^HRs were adjusted for age, gender, smoking status, histology, differentiation and adjuvant chemotherapy or radiotherapy

## Discussion

In the present study, we evaluated the effects of 2 functional SNPs in *HIF1A* on the prognosis of NSCLC patients. Although there was no statistical significant association to be found in the primary analyses, significant associations were observed in the stratified analysis between variant-containing genotypes of SNPs and reduced death or recurrence risk in patients with early TNM stage (I + II) diseases, in patients with early T-stage, and in patients without lymph node involvement (early N-stage). Our data indicated that functional SNPs in *HIF1A* gene had a potential predictive role in prognosis of early stage NSCLC.

Previous study demonstrated that overexpression of HIF1α accelerated glycolysis in A549 NSCLC cells [28]. Elevated level of HIF1α was associated with increased tumor-xenograft growth, whereas inhibition of HIF1 activity remarkably impaired tumor growth in vivo [29, 30]. The specific consequences of increased HIF1 activity, however, varied according to different cell types. In clinical studies focusing on the association between HIF1α expression and prognosis of cancers, the conclusions were contradictory. Volm and Koomagi showed that patients with HIF1α-positive carcinomas had significantly longer median survival time than patients with HIF1α-negative carcinomas [21]. Moreover, Kuo and colleagues investigated the role of 2 non-synonymous SNPs (rs11549465 or Pro582Ser and rs11549467 or Ala588Thr) in coding region of *HIF1A* gene in NSCLC and found that patients carrying T/T genotype of rs11549465 or A/A genotype of rs11549467 had increased cancer risk compared with patients carrying other genotypes [31]. However, the association between the polymorphisms in *HIF1A* gene and NSCLC cancer prognosis is not well investigated so far. Our study indicate that rs2057482 was associated with both OS and RFS in patients with early stage (I + II) NSCLC. Furthermore, we evaluate the effect of rs2057482 and rs2301113 in patients with different T- and N-stages separately. The significant or borderline significant protect effects of variant-containing genotypes of rs2301113 and rs2057482 on OS and RFS were observed only in patients with early T-stage or N-stage diseases, but not in patients with advanced T-stage or N-stage disease. Our findings provide epidemiological evidence that polymorphism in *HIF1A* gene may serve as prognostic factor in NSCLC, especially in early stage disease, although the underlying mechanism needs further investigation.

HIF1 is a transcription factor that controls the expression of more than 40 target genes which encode proteins that play crucial roles in tumor progression, such as vascular endothelial growth factor (VEGF), glucose transporters 1 and 3 (GLUT1, GLUT3), and glycolytic enzymes [32]. In breast cancer, HIF1α overexpression can be detected in ductal carcinoma in situ, the pre-invasive stage at which angiogenesis is first induced [33]. HIF1α was also overexpressed in preneoplastic and premalignant lesions such as colonic adenoma and prostate intraepithelial neoplasia. HIF1α-positive cells were prominent at tumor margins and surrounding areas of neovascularization [34], implying that up-regulation of HIF1α was an early molecular event in carcinogenesis and tumor invasiveness. These features of HIF1α may explain the predictive role of polymorphisms in *HIF1A* gene in early stage of NSCLC. Hypothetically, in advanced NSCLC, the variation in *HIF1A* has some effect on survival, but could not as the independent prognostic factor due to other molecular factors and microenvironmental elements involved in tumor growth. Evidence on this concept is further warranted, and more future research work is needed to elucidate whether our findings can be explained by stage specificity role of *HIF1A* SNPs in tumor progression.

SNP rs2057482 which was significantly associated with NSCLC prognosis is located in the 3′-UTR of the *HIF1A* gene. Previous studies have shown that the polymorphisms in the 3′-UTRs of several genes were associated with miRNA-regulated protein expression by providing mutated binding sites for proteins and microRNAs to alter mRNA stability, or by forming hairpin loop structures to stabilize mRNA and thus slow down degradation [35]. It is assumed that SNP rs2057482 at 3′-UTR might alter *HIF1A* gene expression and subsequently affect survival of NSCLC patients. Further study is needed to verify our assumption.

In summary, as the first study observing the effects of *HIF1A* polymorphisms on NSCLC prognosis, our results strongly suggest that SNPs of *HIF1A* (rs2057482 and rs2301113) are independent prognostic markers for early stage NSCLC patients after surgery. Large-scale studies will be carried out in the future to further validate the findings of the present study.

## Electronic supplementary material

Below is the link to the electronic supplementary material.

Supplementary material 1 (PDF 698 kb)
