# Supplementary material for: Polymorphisms of HIF1A gene are associated with prognosis of early stage non-small-cell lung cancer patients after surgery
Source: Med Oncol. 2014 Feb 25;31(4):877. doi: 10.1007/s12032-014-0877-8 (PMC3971443; doi:10.1007/s12032-014-0877-8)
Supplement: Supplementary file 1 — Supplementary material 1 (PDF 698 kb) [file 12032_2014_877_MOESM1_ESM.pdf]

Medical Oncology

Polymorphisms of HIF1A gene are associated with prognosis of early stage non-small cell lung cancer patients after surgery

Boya Liu[#], Qingchun Liu [#], Yang Song, Xiaofei Li, Yunjie Wang, Shaogui Wan, Zhipei Zhang[*], Haichuan Su[*].

[#]Boya Liu and Qingchun Liu contributed equally to this work.

*Correspondence should be addressed to:

Haichuan Su, M.D., Ph.D.; E-mail: suhc@fmmu.edu.cn.

Zhipei Zhang, M.D., Ph.D.; E-mail: zzpzyy@fmmu.edu.cn.

Supplementary Figure Captions

Supplementary Fig 1 Kaplan-Meier curves of recurrence-free survival by dominant model in patient subgroups with different disease stages

A) The analysis of rs2057482 in patients with early stage disease; B) rs2057482 in patients with advanced stage disease; C) rs2301113 in patients with early stage disease; D) rs2301113 in patients with advanced stage disease

Supplementary Fig 2 Kaplan-Meier curves of recurrence-free survival by dominant model in patient subgroups with different T-stages

A) The analysis of rs2057482 in patients with early T-stage disease; B) rs2057482 in patients with advanced T-stage disease; C) rs2301113 in patients with early T-stage disease; D) rs2301113 in patients with advanced T-stage disease

Supplementary Fig 3 Kaplan-Meier curves of recurrence-free survival by dominant model in patient subgroups with different N-stages

A) The analysis of rs2057482 in patients without lymph node involvement; B) rs2057482 in patients with positive lymph nodes involvement; C) rs2301113 in patients without lymph node involvement; D) rs2301113 in patients with positive lymph nodes involvement

Table S1. Association of *HIF1A* SNPs with clinical outcomes of NSCLC patients

SNP	Genotype	Overall survival			Recurrence-free survival		
		Death/total	HR (95%CI)[a]	*P* value	Recurrence/total	HR (95%CI)[a]	*P* value
rs2057482	CC	110/344	Ref.		154/344	Ref.	
3'-UTR	CT	33/128	0.89(0.60-1.32)	0.551	45/128	0.77(0.55-1.08)	0.132
	TT	5/19	0.61(0.25-1.51)	0.288	8/19	0.73(0.35-1.51)	0.389
	Dominant	38/147	0.84(0.57-1.22)	0.347	53/147	0.77(0.56-1.06)	0.111
	Additive		0.84(0.62-1.14)	0.262		0.81(0.63-1.06)	0.114
rs2301113	AA	72/219	Ref.		101/219	Ref.	
Intron	AC	58/209	0.87(0.65-1.18)	0.442	82/209	0.87(0.65-1.17)	0.371
	CC	18/64	0.82(0.47-1.39)	0.457	24/64	0.72(0.46-1.14)	0.174
	Dominant	76/273	0.84(0.64-1.11)	0.336	106/273	0.83(0.63-1.10)	0.202
	Additive		0.89(0.71-1.13)	0.352		0.86(0.71-1.06)	0.135

Abbreviations: CI, confidence interval; HR, hazard ratio; Ref., reference.

[a]Adjusted for age, gender, smoking status, histology, TNM stage, differentiation, chemotherapy or radiotherapy.

Table S2. Stratified analysis on association of *HIF1A* SNPs with prognosis in NSCLC patients

Variable	rs2057482						rs2301113					
	Death/total		**HR[a] (95%CI)**	**Recurrence/total**		**HR[a] (95%CI)**	Death/total		**HR[a] (95%CI)**	**Recurrence/total**		**HR[a] (95%CI)**
	CC	CT/TT		CC	CT/TT		AA	AC/CC		AA	AC/CC	
**Age**												
≤59	50/173	20/70	0.79(0.46-1.37)	81/173	28/70	0.66(0.42-1.04)	27/109	43/134	1.17(0.71-1.92)	51/109	58/134	0.91(0.62-1.34)
>59	60/171	18/77	0.91(0.52-1.58)	73/171	25/77	1.00(0.62-1.60)	45/110	33/139	0.66(0.42-1.04)	50/110	48/139	0.79(0.52-1.18)
**Gender**												
Female	22/73	9/34	1.09(0.47-2.51)	36/73	12/34	0.84(0.42-1.69)	13/50	18/57	1.08(0.5-2.31)	24/50	24/57	0.93(0.52-1.68)
Male	88/271	29/113	0.79(0.52-1.21)	118/271	41/113	0.75(0.52-1.08)	59/169	58/216	0.79(0.55-1.14)	77/169	82/216	0.81(0.59-1.11)
**Smoking status**												
Never	30/99	14/54	0.75(0.37-1.50)	48/99	21/54	0.60(0.35-1.05)	19/65	25/88	0.80(0.42-1.51)	32/65	37/88	0.72(0.44-1.19)
Ever	80/245	24/93	0.82(0.52-1.30)	106/245	32/93	0.80(0.54-1.20)	53/154	51/185	0.83(0.56-1.23)	69/154	69/185	0.86(0.61-1.20)
**Histology**												
Squamous cell carcinoma	60/201	19/68	0.96(0.57-1.63)	86/201	22/68	0.78(0.48-1.26)	43/131	36/138	0.82(0.52-1.29)	59/131	49/138	0.83(0.57-1.22)
Adenocarcinoma	23/92	9/55	0.72(0.32-1.62)	40/92	15/55	0.58(0.31-1.08)	15/61	17/87	0.82(0.40-1.67)	29/61	26/87	0.61(0.35-1.06)
Others*	27/51	10/24	0.77(0.36-1.69)	28/51	16/24	1.29(0.64-2.59)	14/27	23/48	0.97(0.47-1.98)	13/27	31/48	1.62(0.82-3.23)
**TNM stage**												
**I/II**	60/200	12/86	**0.42(0.22-0.80)**	75/200	22/86	**0.60(0.36-0.97)**	38/130	34/156	0.71(0.44-1.15)	49/130	48/156	0.77(0.51-1.15)
**III**	50/144	26/61	1.54(0.94-2.53)	79/144	31/61	0.93(0.60-1.44)	34/89	42/117	1.07(0.68-1.70)	52/89	58/117	0.92(0.63-1.35)
**Differentiation**												
Well/moderate	66/241	19/102	0.66(0.39-1.12)	99/241	25/102	**0.55(0.35-0.86)**	47/161	38/183	0.69(0.44-1.08)	70/161	54/183	**0.65(0.45-0.94)**
Poorly/undifferentiated	44/103	19/45	0.86(0.48-1.56)	55/103	28/45	1.00(0.61-1.65)	25/58	38/90	0.97(0.58-1.62)	31/58	52/90	1.13(0.72-1.78)
**Adjuvant therapy**												
**No**	33/84	11/43	0.85(0.45-1.60)	39/84	11/43	0.60(0.32-1.15)	22/56	22/71	0.84(0.49-1.45)	26/56	24/71	0.75(0.44-1.28)
**Yes**	77/260	27/104	0.79(0.49-1.28)	115/260	42/104	0.81(0.56-1.18)	50/163	54/202	0.87(0.57-1.31)	75/163	82/202	0.89(0.64-1.23)

Abbreviations: CI, confidence interval; HR, hazard ratio; Ref., reference.

[a]Adjusted for age, gender, smoking status, histology, TNM stage, differentiation, chemotherapy radiotherapy.

Fig S1

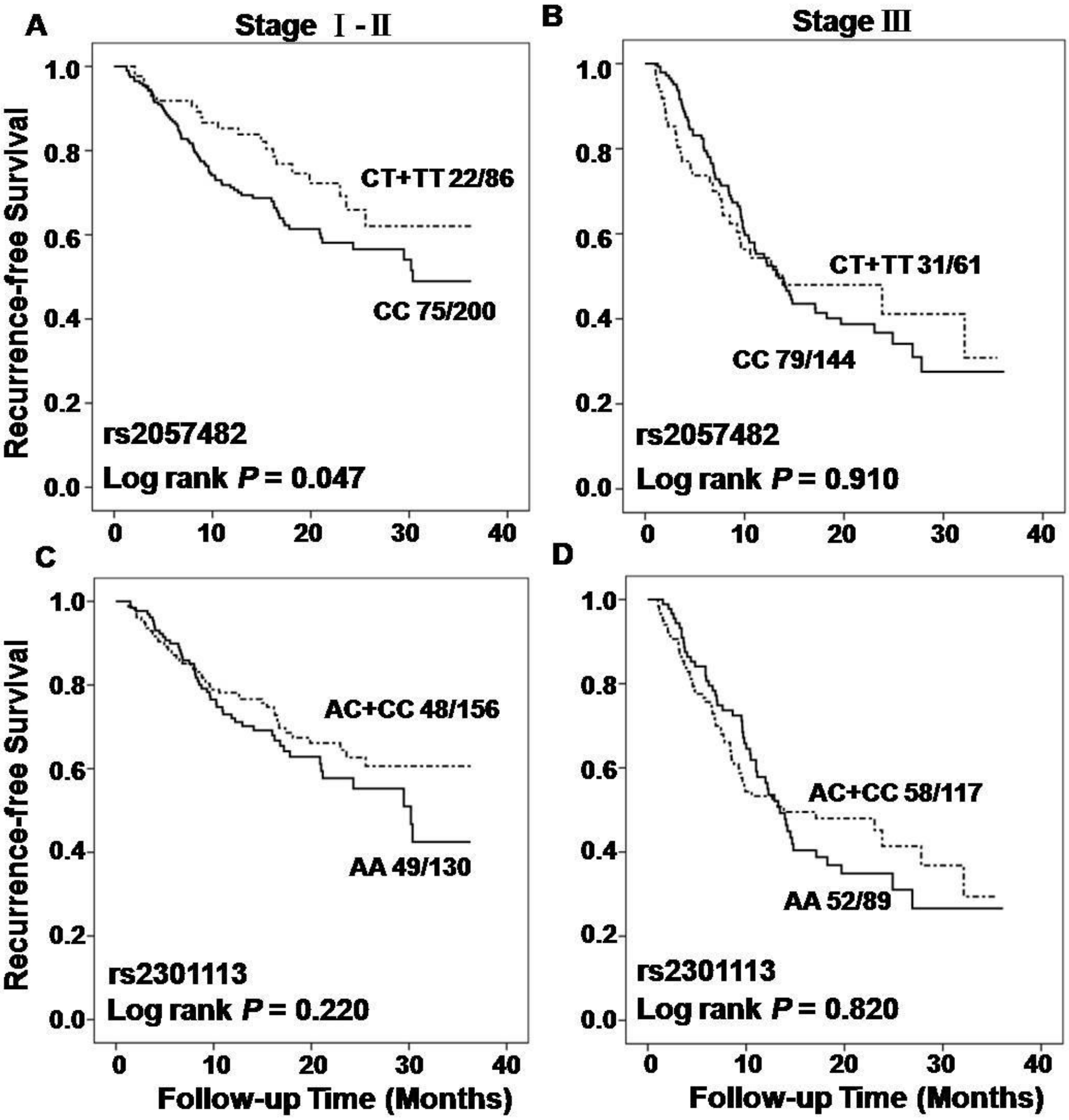
A
Stage Ⅰ-Ⅱ
B
Stage Ⅲ
Recurrence-free Survival
CT+TT 22/86
CC 75/200
rs2057482
Log rank P = 0.047
CT+TT 31/61
CC 79/144
rs2057482
Log rank P = 0.910
C
D
AC+CC 48/156
AA 49/130
rs2301113
Log rank P = 0.220
AC+CC 58/117
AA 52/89
rs2301113
Log rank P = 0.820
Follow-up Time (Months)
Follow-up Time (Months)
1.0
0.8
0.6
0.4
0.2
0.0
0
10
20
30
40

Fig S2

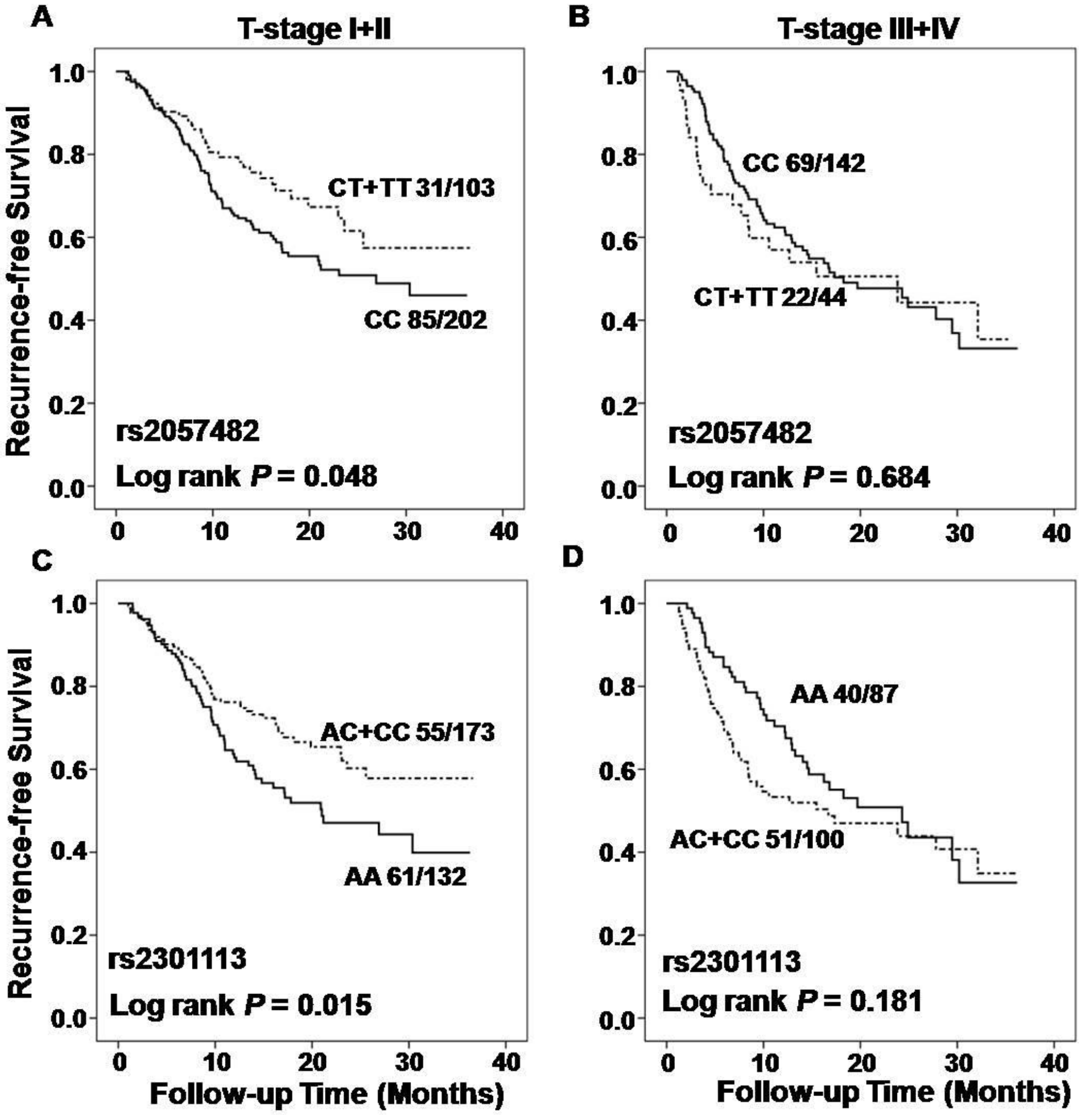
A
T-stage I+II
Recurrence-free Survival
CT+TT 31/103
CC 85/202
rs2057482
Log rank P = 0.048
B
T-stage III+IV
CC 69/142
CT+TT 22/44
rs2057482
Log rank P = 0.684
C
Recurrence-free Survival
AC+CC 55/173
AA 61/132
rs2301113
Log rank P = 0.015
Follow-up Time (Months)
D
AA 40/87
AC+CC 51/100
rs2301113
Log rank P = 0.181
Follow-up Time (Months)

Fig S3

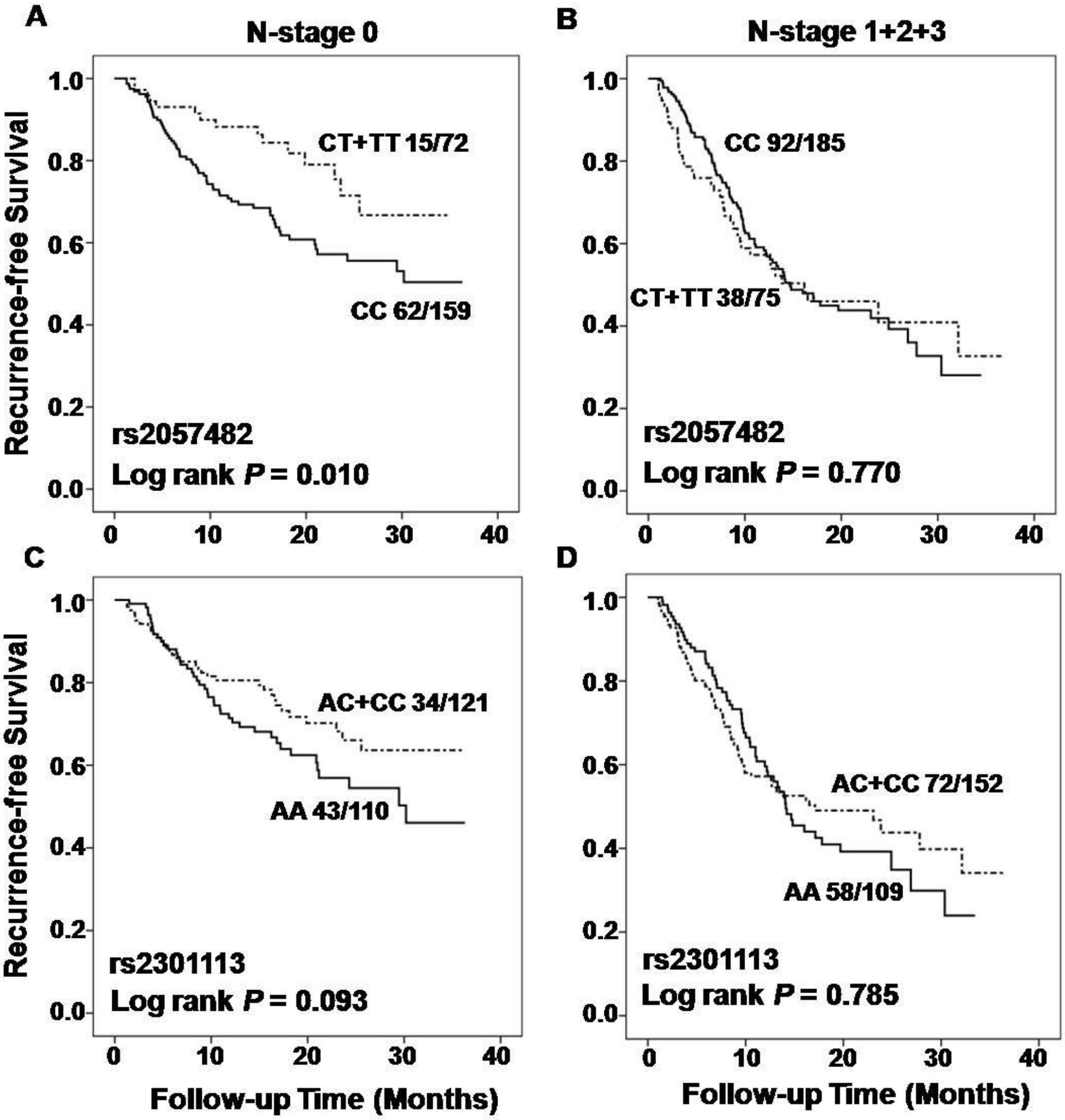

A
N-stage 0
B
N-stage 1+2+3
Recurrence-free Survival
1.0
0.8
0.6
0.4
0.2
0.0
CT+TT 15/72
CC 62/159
rs2057482
Log rank P = 0.010
CC 92/185
CT+TT 38/75
rs2057482
Log rank P = 0.770
0
10
20
30
40
C
D
AC+CC 34/121
AA 43/110
rs2301113
Log rank P = 0.093
AC+CC 72/152
AA 58/109
rs2301113
Log rank P = 0.785
Follow-up Time (Months)
